# A Model for Disentangling Dependencies and Impacts among Human Activities and Marine Ecosystem Services

**DOI:** 10.1007/s00267-020-01260-1

**Published:** 2020-02-27

**Authors:** Andreas Bryhn, Patrik Kraufvelin, Ulf Bergström, Max Vretborn, Lena Bergström

**Affiliations:** 1 Swedish University of Agricultural Sciences, Department of Aquatic Resources, Institute of Coastal Research, Skolgatan 6, 74242 Öregrund, Sweden; 2 Swedish Agency of Water and Marine Management, Gullbergs Strandgata 15, 41104 Göteborg, Sweden

**Keywords:** Assessment model, Ecosystem services, DAPSIR, Ecosystem-based management, Marine fisheries, Marine tourism

## Abstract

Understanding and communicating the links among human activities and marine ecosystem services are fundamental for ecosystem-based management, which aims at attaining ecological, economic and social sustainability in the use of our seas. Relationships are typically complex and may differ between geographic areas. Here, an assessment model that combines available quantitative, semi-quantitative and qualitative information, rooted in the DAPSIR (Driver—Activity—Pressure—State—Impact—Response) framework and assessment requirements of the EU Marine Strategy Framework Directive, is developed and applied. Focusing on Swedish marine waters, major human activities at sea are evaluated in relation to their dependencies and impacts on the status of marine ecosystem services. This case study is a consensus assessment based on evaluation of available literature and data. By relating degrees of dependencies and impacts to values of different economic sectors, discrepancies among sectors with respect to their impact versus their monetary value can be identified. In our case, commercial fishing depends on and influences a wide range of ecosystem services, while other sectors, such as shipping, depend little on marine ecosystem services. At the extreme end of the range, pressures from human activities in the past, such as historical nutrient emissions, still have prominent influence on ecosystem services today, entailing considerable losses. Marine tourism and commercial fishing show similar dependencies on ecosystem services, but tourism has a clearly lower impact on ecosystem services and a higher monetary value. The model may serve as a useful tool for communicating and guiding priorities in integrated environmental management and maritime spatial planning.

## Introduction

Human use of marine waters and resources has strongly altered the structure and function of many marine ecosystems worldwide (Halpern et al. 2008, 2015; Rocha et al. 2015; Selim et al. 2016; Österblom et al. 2017). Impacts have been manifested as various adverse environmental effects, such as fish stock declines or collapses (Worm 2016), loss of biodiversity (Mazor et al. 2018), eutrophication (Schernewski et al. 2015), harmful levels of toxic pollutants (Carlsson et al. 2016), littering (Bergmann et al. 2015) and depletion of valuable habitats (Airoldi and Beck 2007). Thus, human activities also cause effects on marine ecosystem services; i.e., the benefits that people and societies receive from marine ecosystems, and which concurrently serve as preconditions for many human activities related to the sea (World Resources Institute 2005; Barbier 2017). The connection between the state of human activities and ecosystem services (Costanza et al. 2014; Boumans et al. 2015; Anderson et al. 2016) is especially evident in coastal areas, which contain some of the highest valued ecosystem services globally (Costanza et al. 1997, 2014; de Groot et al. 2012; Culhane et al. 2018), while often facing extensive pressure from human activities (Lotze et al. 2006; Elliott et al. 2018).

Importantly, ecosystem services are produced in the dynamic and multi-faceted interface between social and ecological systems (Bennett et al. 2015). Ecosystem components, such as species and habitats, are the basis of ecosystem functions (Palumbi et al. 2009; Ceballos et al. 2015; Hautier et al. 2015), which maintain ecosystem services and may also induce positive impacts on human well-being (Sandifer et al. 2015). Hence, explicit consideration of ecosystem services in environmental management and spatial planning can be expected to lead to improved long-term societal and environmental outcomes (Arkema et al. 2015). In this context, it is imperative for management to consider in which ways species and habitats, the functions they provide, and hence the flow of ecosystem services, can be affected by various human activities (Giakoumi et al. 2015; Mach et al. 2015). As ecosystem services are associated with high societal values (monetary or non-monetary), the consideration of these linkages may, further, support the bridging of different management perspectives, and eliminate or dampen conflicts between development and environmental protection (Balmford et al. 2002; de Groot et al. 2010).

Analyses of ecosystem services are increasingly included in environmental management, although hitherto at a much more limited extent for marine systems than for terrestrial environments (Liquete et al. 2013; Inácio et al. 2018; Schernewski et al. 2018). Among the marine examples, the global Millennium Ecosystem Assessment (World Resources Institute 2005) identified fishing as the most prominent driver of changes in ecosystem services, while Rocha et al. (2015) highlighted excessive anthropogenic nutrient load leading to eutrophication and hypoxia as a major determinant of marine ecosystem functioning and services. Martin et al. (2018) made a qualitative investigation of connections between human activities and ecosystem services in relation to coastal nutrient management in Massachusetts, USA, and found rather diverse effects on ecosystem services depending on which human activity was studied. In the Baltic Sea, Inácio et al. (2018) developed an assessment tool for marine ecosystem services and applied it to conditions in two coastal lagoons with results differing strongly between the lagoons. Furthermore, there may be a considerable spatial mismatch between human impacts, ecosystem functioning, and marine ecosystem services (Lindegren et al. 2018), and non-linear responses are common (Hunsicker et al. 2015). Thus, while analyses of how impacts of human activities affect ecosystem services globally are vital (Costanza et al. 2014; Lindegren et al. 2018), they will inevitably be connected to considerable data and assessment challenges and a high level of site-specificity and context-dependency (Kok et al. 2017).

**Fig. 1:**
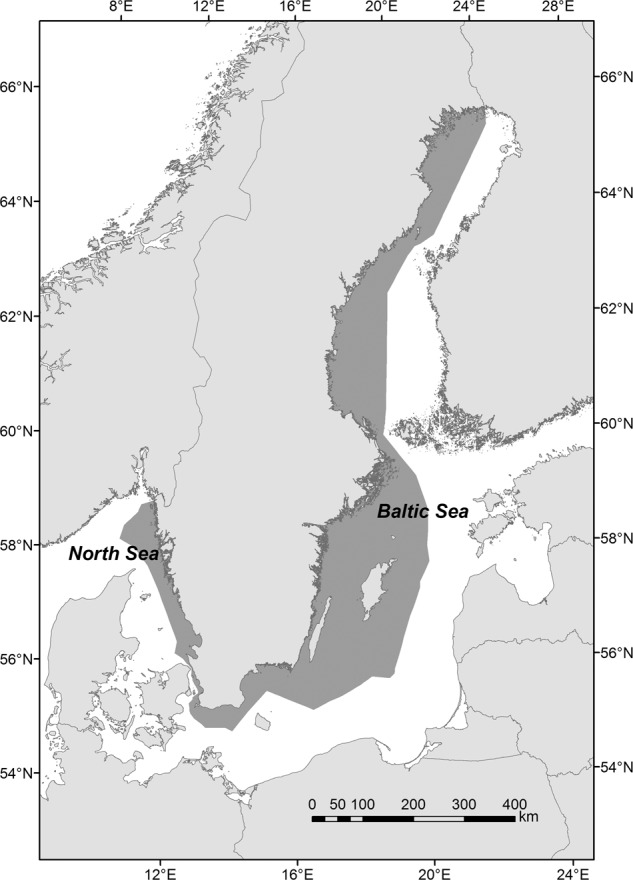
The Swedish marine economic zone (dark grey colour) in Northern Europe, covering a substantial part of the Baltic Sea as well as the easternmost part of the North Sea

For policy-makers, managers and stakeholders, it may be challenging to understand and communicate ecosystem services and associated analyses from an integrated perspective (Beaumont et al. 2007). To address these challenges, this study presents an assessment model for exploring, comparing and communicating dependencies and impacts of human activities on marine ecosystem services over multiple sectors at an overarching policy level. The assessment model addresses three major questions: (1) to what extent are marine ecosystem services affected by different human activities, (2) to what extent are different human activities dependent on specific marine ecosystem services and (3) how do these relationships compare with the economic performance of different sectors of sea use. The assessment model is applied in an example from Swedish coastal and marine waters located in Northern Europe, covering parts of the western and northern Baltic Sea and the eastern North Sea (Fig. 1; see motivation for selecting the study area in the following section). The assessment model connects to recurrent policy practice in relation to the EU Marine Strategy Framework Directive, where addressing the state of the environment in relation to our use of marine waters and impacts on ecosystem services and human well-being is a key element (EC 2008, 2017; Elliott et al. 2017), but it is also of relevance for other situations when there is a need to address multidimensional interlinkages between economic and environmental aspects.

## Materials and Methods

The study area encompasses Swedish coastal and marine waters (Fig. 1), which represent a rather diverse mix of urban and industrial areas, popular recreational areas, sparsely populated regions, as well as open marine waters with marine shipping routes and various levels of fishing pressure. The approach used in this study is exemplified at this overarching scale, as governmental bodies responsible for marine environmental management often have geographically wide (national) mandates (Schreiber and Linke 2018; Haight et al. 2019), and there is therefore a need for analyses and syntheses of information across sectors at this scale.

**Fig. 2:**
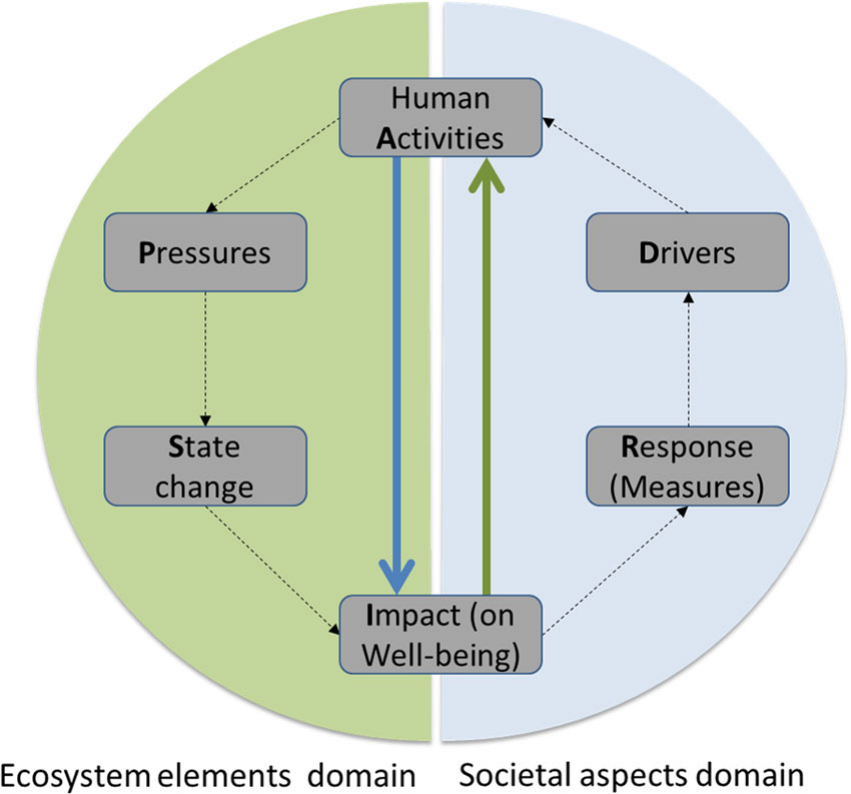
Illustration of how the DAPSIR framework is applied in this work, focusing on the reciprocal relationships between human activities and impacts (ecosystem services), as highlighted by the thick vertical arrows

Our assessment model aligns with the generic Driver—Activity—Pressure—State change—Impact—Response (DAPSIR) framework (Fig. 2), which is widely used in environmental management as a way to describe causal relationships between society and the environment (Atkins et al. 2011; Patrício et al. 2016; Martin et al. 2018). The DAPSIR framework is useful to support the shared understanding among actors, including representatives of science, policy-makers, managers and stakeholders, and has subsequently been developed into various adaptations (Patricio et al. 2016; Elliott et al. 2017). The basic framework outlines the causal links between drivers, activities, pressures, environmental status, impacts, and responses in a management setting (Atkins et al. 2011). Figure 2 illustrates the focal aspects for the purposes of this work: the human activities (A), which may cause pressures on the environment and subsequent effects on ecosystems, as illustrated in the left-hand side of the cycle, and the impacts (I) on ecosystem services and human well-being, which may occur as a result. The results are relevant for the development of management responses, by providing information on which measures may be required, and potentially for guiding policies aimed at changing the drivers of human activities. Hence, analyses based on the DAPSIR framework not only provide information about how different activities may influence the marine environment, including the provisioning of ecosystem services, but also about how human activities depend on the environment and the likely associated incentives to change from status quo.

The first two steps in the evaluation correspond to assessing the likely influence of human activities on the status of ecosystem services, and vice versa, as illustrated by the vertical thick arrows in Fig. 2. Thus, the complete causal impact chain (human activities—pressure—state—impact) is not central, although this can be included by embedding more detailed information from cascade models when available (Haines-Young and Potschin 2010; La Notte et al. 2017). For the purposes of this study, the estimations were performed as an internal consensus expert evaluation with structured elicitation supported by a literature review (see McBride et al. 2012; Smith et al. 2015; more information below).

The matrix for evaluation encompassed a wide range of human activities and marine ecosystem services (Tables 1 and 2). In all, 21 human activities were assessed, representing ongoing activities in Swedish seas today and using terminology and classification aligned with Annex III in EC (2017) (Table 1). In addition, five still enduring ambient pressures that are not directly connected to ongoing human activities were included. Most prominently, sedimentary internal loading of nutrients and contaminants emanating from past anthropogenic emissions still give rise to pressures in the marine environment in the assessed area (HELCOM 2018). Furthermore, environmental pressures related to climate change were included without being linked to certain ongoing human activities. For this, a decrease in surface water salinity, an increase in water temperature and an increase in carbon dioxide that may cause acidification are used; see BACC II author team (2015). Several human activities in the study area are likely to exacerbate global climate change, but a vast majority of the impact is due to past and globally occurring activities (Schuur et al. 2015).

**Table 1:** Human activities considered in the study, as well as pressures from past activities

Activity
Land claim
Restructuring of seabed morphology, including dredging and depositing of materials
Extraction of minerals (rock, metal ores, gravel, sand, shell)
Renewable energy generation (wind, wave and tidal power), including infrastructure
Nuclear power (uptake and discharge of cooling water)
Transmission of electricity and communications (cables)
Fish and shellfish harvesting (professional)
Hunting and collecting for other purposes
Aquaculture — marine, including infrastructure
Agriculture
Forestry
Transport — infrastructure
Transport — shipping
Urban uses
Industrial uses
Waste treatment and disposal
Tourism and leisure infrastructure (including marinas)
Tourism and leisure activities (including boating)
Fish and shellfish harvesting (recreational)
Security/defense (military)
Research, survey and educational activities
**Pressures from past activities**
Eutrophication legacy
Contamination legacy
Climate change carbon dioxide (CO_2_)
Climate change temperature
Climate change salinity

**Table 2:** Marine ecosystem services in the Swedish marine economic zone (remade from Bryhn et al. 2015)

Regulating and supporting	Provisioning
RS1: Biogeochemical cycling	P1: Food
RS2: Primary production	P2: Raw material
RS3: Food web dynamics	P3: Genetic resources
RS4: Biodiversity	P4: Chemical resources
RS5: Habitat	P5: Ornamental resources
RS6: Resilience	P6: Energy
RS7: Climate and atmospheric regulation	
RS8: Sediment retention	**Cultural**
RS9: Regulation of eutrophication	C1: Recreation
RS10: Biological regulation	C2: Aesthetic values
RS11: Regulation of toxic substances	C3: Science and education
	C4: Cultural heritage
	C5: Inspiration
	C6: Natural heritage

The letters preceding each ecosystem service indicate their categorization into either regulating and supporting (RS), provisioning (P), or cultural (C) ecosystem services

With regard to ecosystem services, a multitude of classifications and typologies exist (e.g. Böhnke-Henrichs et al. 2013; Hasler et al. 2016; Ivarsson et al. 2017; Maes et al. 2018). The diversity partly reflects that this is a field in development, but partly also the need for adapting functionally operative systems for different purposes. However, many of the existing typologies are still comparable at some level of classification. For the purposes of this work, a Swedish national marine typology developed by Garpe (2008), and further elaborated by Bryhn et al. (2015) was used. This typology includes 23 ecosystem services distributed over three groups: regulating and supporting, provisioning as well as cultural ecosystem services (Table 2).

In the present evaluation, the first two steps were performed by authors AB, PK, UB and LB, who were tasked to synthesize the current level of knowledge in the field for the concerned geographical area. The four academic experts were selected by the Swedish Agency of Marine and Water Management (SwAM). The newest available assessment sources were identified as SwAM (2012, 2018a–d), Bryhn et al. (2015), OSPAR (2017), and HELCOM (2018), which were supplemented with results from research papers. Referring to areas of expertise, the group represented research, environmental monitoring and assessment on ecosystem-based management and ecosystem services (AB, LB), impacts of human activities including in particular fishing (UB, AB), toxic pollutants (AB) eutrophication (AB, PK), non-indigeneous species (PK), habitat deterioration (PK, UB) and marine renewable energy (LB) as well as marine ecology and food webs including ecosystem functioning, green infrastructure and restoration (PK, UB, LB).

### Step 1. Impacts of Human Activities on Marine Ecosystem Services

Impacts from each of the human activities on each of the ecosystem services were estimated applying an ordinal classification scale of 0–4, where 4 represents the highest impact and 0 no impact, focusing on negative impacts. Potentially positive impacts (e.g., added hard surfaces in connection with shipwrecks, coastal construction or when deploying artificial reefs) were regarded as very minor at the large scale considered in this study and were therefore not assessed.

The scores were assigned in a matrix covering all combinations of the assessed human activities and ecosystem services (Supplement 1), considering information on the intensity of pressure caused by each activity as well as its geographical prevalence in the assessed area. Impacts from a human activity causing an intense pressure but with a relatively limited spatial extent attained a lower score than a less intense pressure acting over a wider area. For instance, marine aquaculture may cause relatively strong pressures in the form of physical habitat loss or nutrient inputs, but has a restricted geographical extent in Sweden (Statistics Sweden 2017). Conversely, shipping may be attributed to pressures of relatively low intensity (underwater noise, wakes, nutrient input, littering, or potential spread of non-indigenous species) but is much more widespread (Klusek 2016).

In a first step, author PK provided individual scores to all combinations of human activities and ecosystem services based on the above indicated sources. Following this initial scoring, all assessors (AB, PK, UB, LB) gathered, scrutinized the matrix, and wherever differing views appeared, re-evaluated available evidence in order to reach consensus. When doing so, the results were scrutinized from both of two perspectives: assessing each human activity in relation to all ecosystem services, as well as each ecosystem service in relation to all human activities. The purpose of this procedure was to screen for potential inconsistencies in the scaling of scores for different human activity and ecosystem service combinations, and scores were adjusted when motivated.

### Step 2. Dependencies of Human Activities on Ecosystem Services

For estimating the dependencies of each of the human activities on each of the ecosystem services (Tables 1, 2), assessment steps recommended by Ivarsson et al. (2017) were considered. The resulting scores were compiled in a second matrix (Supplement 2). The evaluation built on previous work by SWaM (2012), within which a lower number of human activities were evaluated using binary (0 and 1) scoring. However, in the current study, five ordinal classes (0–4) were applied, where the highest score (4) corresponds to the highest dependency. The evaluation process followed the same procedure as in the assessments of impacts on ecosystem services (Step 1): Initial scores provided by author PK based on agreed criteria, followed by group discussion, evaluation of results, and scrutinization in relation to other combinations of human activities and ecosystem services, to reach a consensus score. In this assessment, pressures from past activities (Table 1) were assigned values zero, as being independent of current marine ecosystem services.

Hence, reflecting the aim to reach a generic consensus assessment, only integers were reported in the results. Uncertainty was not assessed since the focus of the study was on developing the model for assessing dependencies and impacts among human pressures and ecosystem services. Adding an uncertainty estimation of the scores is a clear topic for later refinement of the method but will require a separate consideration given the broad and overarching assessment scale.

In a final step, the scores within each of the two matrices were summed up for both human activities and ecosystem services (rows and columns in each matrix) to attain an overall measure of their cumulative impact/dependency on each other (Supplements 1, 2). In the aggregated scores, all ecosystem services categories and all activity categories were given equal importance weighting. The process to develop consensus scores in the two assessments required five dedicated meetings plus shorter follow-ups by short meetings or e-mail.

### Step 3. Combining the Evaluation Outcomes

In a third step, information on monetary value was collected for all activities for which such data were available. The monetary value estimates comprised the value added by specific marine sectors, based on national statistics using the Statistical Classification of Economic Activities in the European Community (NACE) codes for each activity according to Statistics Sweden (SwAM 2017), with the addition of unpublished data on recreational fishing from SwAM. In our applied example, this part of the analysis included some aggregated activity categories compared with those applied in steps 1 and 2, depending on the available aggregations for data on economic values. Here, we had economic data for marine tourism, and importance scores for marine tourism were obtained by taking the maximum values of the activities categories ‘tourism and leisure infrastructure’ and ‘tourism and leisure activities’. This was regarded among the assessors as a suitable precautionary method.

To indicate the relative dependency and potential negative impact on marine ecosystem services of the different human activities, the tallied total scores from the evaluations attained in steps 1 and 2 were used. The assessment results were combined by plotting the sum of scores for the dependency of each human activity on ecosystem services versus the sum of scores for their impacts on the same, to reflect the extent of interdependencies between human activities and ecosystem services.

## Results

**Fig. 3:**
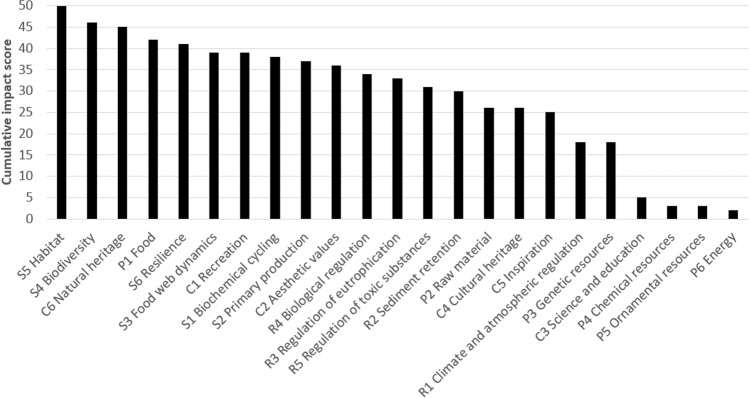
Rank order of marine ecosystem services in relation to how much they are impacted by human activities, according to the applied expert judgement

**Fig. 4:**
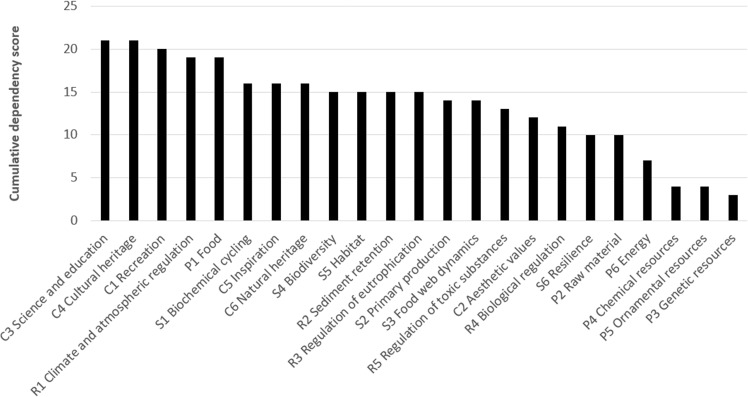
Rank order of marine ecosystem services in relation to their summed importance for human activities, according to the applied expert judgement

**Fig. 5:**
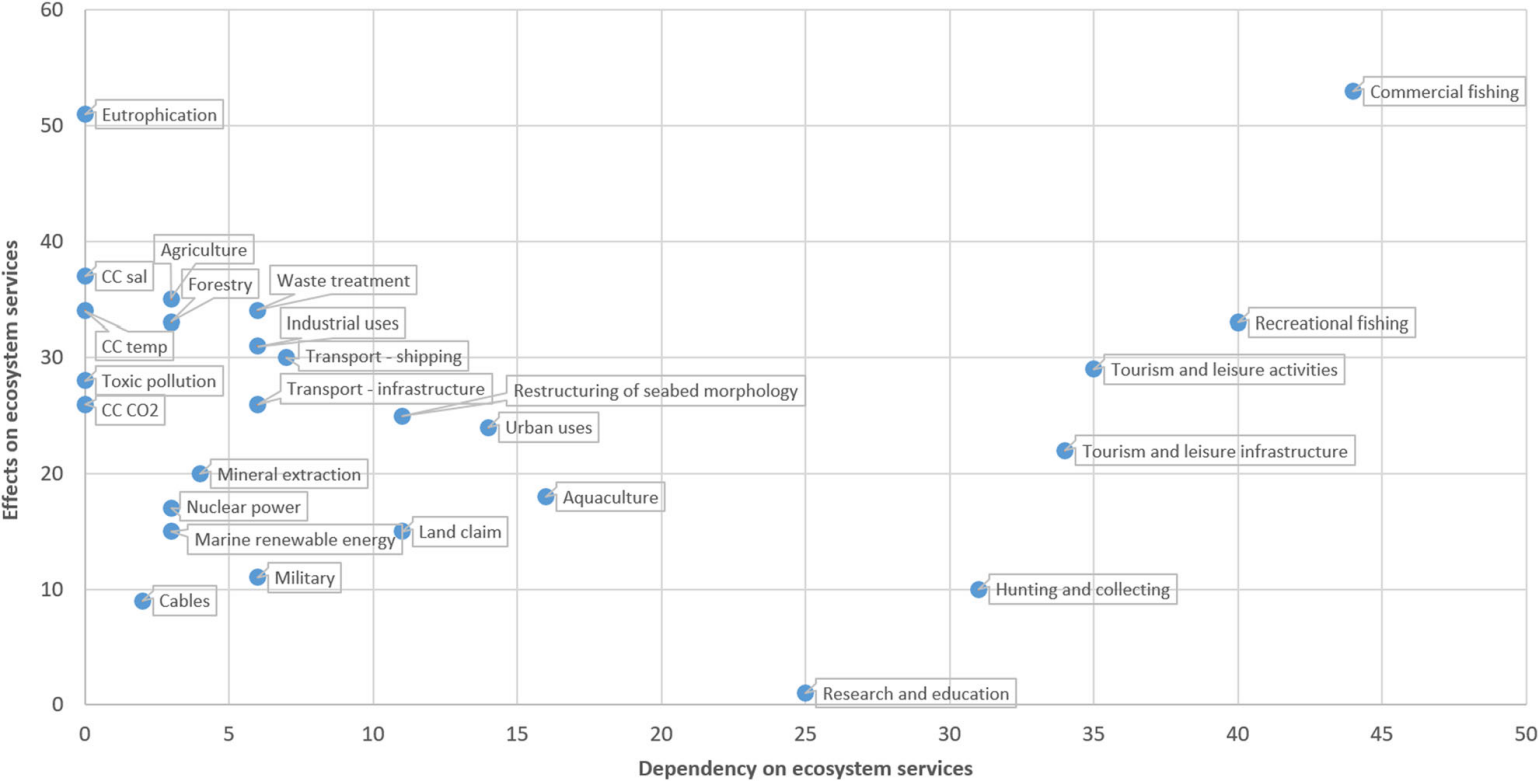
Relationship between the dependencies on ecosystem services of marine human activities (*x*-axis) and the impacts of the same human activities on ecosystem services (*y*-axis), using the described expert evaluation method. Pressures from past emissions are not dependent on current marine ecosystem services and score 0 on the *x*-axis (highlighted in yellow)

**Fig. 6:**
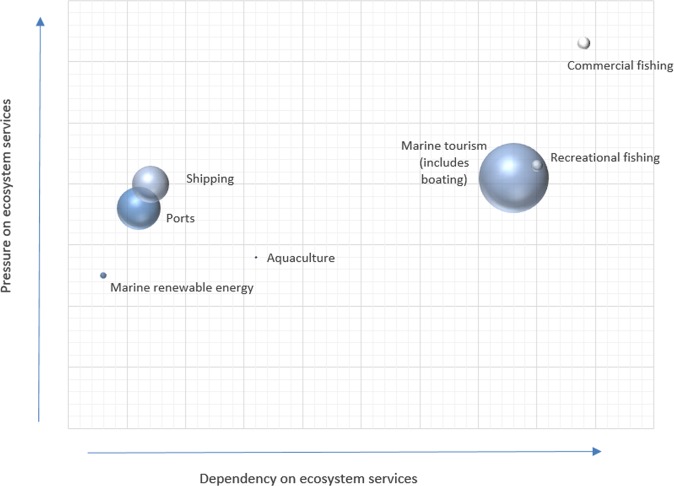
The dependency of human activities on marine ecosystem services (x-axis) and the impact of human activities on ecosystem services (*y*-axis). The size of the bubbles represents their monetary value added. The total monetary value of all the sectors represented in the diagram is 1.5% of the Swedish gross domestic product. Note that the activity categories are more aggregated in this figure than in other figures, depending on the data availability on economic values. For data, see Appendix (Table 3)

The applied results for the Swedish coastal and marine area are presented in Figs 3–6 and in the Supplement. The expert assessment shows the relative extent to which various human activities potentially impact on the status of ecosystem services and are dependent on these in Swedish coastal and marine areas. According to this evaluation, commercial and recreational fish and shellfish harvesting, agriculture and waste treatment and disposal, out of the examined activities, had the highest ratings regarding influence on ecosystem services. However, the background pressures eutrophication due to past activities, and climate change related temperature increase and salinity decrease were also identified as important (Supplement 1). Other activities had a relatively smaller impact on ecosystem services in this assessment. The most strongly impacted ecosystem services were identified among the regulating and supporting services, ‘Habitat’ and ‘Biodiversity’, but for instance the provisional service ‘Food’ and the cultural service ‘Recreation’ were also relatively highly rated (Fig. 3; Supplement 1). These represent ecosystem services that are impacted by either widely distributed pressures or by several pressures in combination, reflecting cumulative impacts. Other ecosystem services were primarily affected by fewer activities or to a lesser spatial extent (e.g. ‘Chemical resources’, ‘Ornamental resources’, and ‘Energy provision’).

Overall, the ecosystem services with strongest connection to human activities (Fig. 4, Supplement 2) were identified as ‘Science and education’, ‘Cultural heritage’ and ‘Recreation’ (cultural services), as well as the provision of food (provisioning service) and some regulating or supporting services (‘Biogeochemical cycling’, ‘Biodiversity’ and ‘Habitat’). At the other end of the scale, human activities were assessed as being little dependent on chemical, ornamental and genetic resources (provisional services; Fig. 4). Most of the individual scores (Supplement 2) were uncontroversial among the assessors, while a few were subject to longer discussion, reflecting lower certainty, such as the impact of commercial fishing on the ecosystem service ‘Raw material’. In this part, the discussion led to considering fish for industrial purposes as an important part of raw material from the sea (*sensu* Garpe 2008 and Bryhn et al. 2015). Marine renewable energy (wind and wave power) were not included in the ecosystem service ‘Energy’, since Garpe (2008) and Bryhn et al. (2015) only include energy based on biogenic material in this ecosystem service category.

Considering individual human activities, ‘commercial and recreational fish and shellfish harvesting’, ‘tourism and leisure activities and infrastructure’, ‘hunting and collecting’, as well as ‘scientific and educational activities’ scored as being the most dependent on ecosystem services (Supplement 2). The results reflect that the extraction and harvesting of natural resources was noted as being highly dependent on various regulating or supporting and provisioning ecosystem services, and activities related to tourism additionally on cultural ecosystem services. Many human activities, including ‘shipping’, ‘agriculture’, ‘forestry’, ‘transmission (cables)’, scored very low regarding dependency on marine ecosystem services (Supplement 2).

The relative position of all studied human activities in relation to their tallied estimated dependency and impact on ecosystem services, respectively, is shown in Fig. 5. Figure 6, again, shows the same result when information on monetary estimates of human activities is added where available. For example, ‘marine tourism’^1^ and ‘commercial fishing’, which were both assigned as being highly dependent on ecosystem services, differed in that ‘marine tourism’ was identified as having lower impact on ecosystem services than ‘commercial fisheries’, but also a higher monetary value (indicated by the size of the bubble). Transportation related activities (‘shipping’ and ‘ports’), which were estimated as having similar level of impact on ecosystem services as ‘marine tourism’, clearly differed from the latter by having a lower dependency on ecosystem services. The monetary value was also lower than for ‘marine tourism’ (Fig. 6).

## Discussion

This study investigated and quantified the dependency and impact of human activities on ecosystem services using a structured assessment model to make use of available information for different sectors together. Comparative investigations of the impact of human activities on marine ecosystem services have been undertaken previously (e.g. World Resources Institute 2005; Giakoumi et al. 2015; Rocha et al. 2015), but have not so far integrated the analyses with an assessment of the dependency of human activities on marine ecosystem services (see, however, SWaM 2012). Ivarsson et al. (2017) developed a methodology for the latter kind of analysis, but did not proceed to testing it. Their suggested approach was considered in the development of our study, and was adapted to suit our data. Ivarsson et al. (2017) suggested a binary scoring system (0 or 1) while the present study used a 0–4 scoring system to provide a more fine-scale assessment of the impact on ecosystems services from the various activities. Using the former approach would probably yield more conspicuous results for activities with low dependencies on marine ecosystem services, such as agriculture, forestry and marine renewable energy.

According to the applied evaluation, commercial fishing and eutrophication legacies were the activities or pressures that affected marine ecosystem services most fundamentally (Fig. 5; see also Rocha et al. 2015; Barbier 2017). Commercial fishing had an exceptional position in being both highly impacting and highly dependent on ecosystem services. In all, only a few of the assessed activities were identified as having a high dependency on ecosystem services, these being mainly related to the extraction of living marine resources (fishing, hunting and collecting for commercial or recreational purposes) or tourism and recreation. Most activities had low dependencies on marine ecosystem services, including e.g. ‘Urban uses’, construction works, energy production and transport. These activities might be expected to take place at a similar extent regardless of the status of the marine environment, or be little influenced by a change in state, but have an effect on other human activities via their impacts on ecosystem services. Eutrophication legacies, which had among the largest impacts on ecosystem services, invoke only costs and no benefits (Fig. 5). The current loss of benefits due to eutrophication has been estimated at the scale of billions of Euros annually for the Baltic Sea region (HELCOM 2018).

In all, the results (Figs 5, 6) indicate that several human activities exert pressures on the marine environment and ecosystem services, but that only some of them would benefit substantially from a better state. The results thus suggest a lack of direct incentives for environmental adaptation for several activities. In fact, even for activities that are both dependent on and clearly affecting ecosystem services, such as commercial fisheries, incentives for voluntary environmental adaptation to favour long-term benefits have often been found inadequate (Grafton et al. 2016). However, as commercial fisheries would benefit from sustainable fish stocks and healthy habitats in the multiannual perspectives, their value should be expected to increase by improved management instruments (Squires and Garcia 2018). As marine ecosystem services are often highly valued in Swedish waters and in the Baltic Sea in general (Cole and Moksnes 2016; Oinonen et al. 2016; HELCOM 2018; Nainggolan et al. 2018), the lack of direct incentives for some sectors and actors to safeguard ecosystem services may necessitate well-designed fiscal mechanisms to enable environmental actions (Nainggolan et al. 2018). For wide-ranging activities and impacts, such as in the cases of eutrophication legacies, contamination legacies (toxic pollution), climate change and offshore commercial fishing, internationally coordinated actions are crucial also for the national management level (Nainggolan et al. 2018). The national scale was applied in the current assessment as it was considered the most useful one to support current environmental policy requirements (EC 2008, 2017). Although the assessment hence provides an overarching view, it should be recognized that there is substantial spatial variability at a more detailed scale, which the current approach does not aim to depict. For instance, ecosystem services related to marine recreation are expected to be largely confined to coastal areas, whereas commercial fishing yields higher market values in the open sea than in coastal areas of Sweden. Regarding differences among geographical areas, coastal recreation is more prominent in the densely populated southern parts of Sweden, for instance in the Bohuslän and Stockholm archipelagos (Jaccopucci and Gunvaldsson 2013), than in the north. For analyses to provide such differentiation, additional spatially explicit evaluations are required.

Considering the economic value of the activities (Fig. 6), it can be seen that marine tourism had a much higher monetary value nationally than fisheries. As tourism was also assessed as having a clearly lower impact on ecosystem services, a possible implication could be that marine tourism is looked upon as having higher priority than commercial fishing, for both economic and environmental reasons. However, such prioritization must also be put in the context of other societal needs, including food security (Pihlajamäki et al. 2016). The high ranking of marine tourism regarding dependency on ecosystem services, is however, well supported by literature. Naturalness is often ranked highly in importance among marine tourists (Ryan and Page 2011; Jacobsen and Tømmervik 2016), reflecting that well-functioning ecosystems are appreciated (Jacobsen and Tømmervik 2016). For instance, healthy habitats may be a key reason for visiting a certain area (Ryan and Page 2011) while high prevalence of marine litter, harmful algal blooms and other outbreaks or invasions, conversely, may deter visitors (Krelling et al. 2017; Groeneveld et al. 2018).

The DAPSIR framework used in this study to define the evaluation context was primarily useful to structure the setting of scores to address impacts from human activities on ecosystem services (and vice versa), and to ensure that the steps of the assessment were presented in an easily understandable and transparent way, supporting communication and discussion of results. Hence, this study provides an example of how the interrelationships between activities and ecosystem services can be assessed to guide management priorities and facilitate communication among stakeholder groups in support of an ecosystem-based approach. Including assessment uncertainties would be a desirable further development of the study (see e.g. Trochta et al. 2018), as would engaging a broader panel of experts to perform the assessments. These shortcomings underscore the importance for managers to interpret our results with care.

As an alternative to consensus assessment, as was the applied procedure here, it may be possible in future work to include information on differences in assessed scores between different experts, since not having to reach consensus would require less time and effort from each assessor. However, the consensus process also led to a more well-informed and balanced assessment in our view, as existing studies and results were available to the same level to all assessors. Having experts contributing with an individual set of scores each would have changed the results at some points, but as there was not much disagreement about the scores among the authors, so this alternative method would only have generated minor differences compared with the current assessment. Comparing results for different ecological settings would also be valuable, such as extending the assessment to other sea areas than the Swedish marine waters. For example, in economies with a higher dependency on the fisheries sector (in Sweden, fisheries and aquaculture account for <0.1% of the gross domestic product; OECD 2014) could be expected to yield considerably differing results.

Importantly, the assessment model developed and used here provides a tool for communication among and within different groups of researchers, managers and stakeholders, and illumination of potential tradeoffs between different human activities and marine ecosystem services. Stakeholder involvement is increasingly advocated as a key for successful implementation of ecosystem-based management (Bryhn et al. 2017; Schreiber and Linke 2018). Ecosystem services may be difficult to understand and communicate (Beaumont et al. 2007) and the present approach may serve as one example of how to make the role of ecosystem services more easily understood, more widely discussed and analysed. This could lead to a more holistic and multidisciplinary management, highlighting both ecosystem and societal aspects of the marine environment.

### Supplementary information

Supplement 1

Supplement 2

## Appendix

**Table 3:** Value added from 7 different categories of human activities and their assessed impacts and dependency on marine ecosystem services

	Impacts on ecosystem services	Dependency on ecosystem services	Value added (Swedish crowns, SEK, in thousands)
Commercial fisheries	53.0	44.0	650,000
Ports	26.0	6.0	7,447,000
Aquaculture	18.0	16.0	16,588
Marine renewable energy	15.0	3.0	140,910
Shipping	30.0	7.0	5,365,000
Marine tourism^a^	31	38	19,235,891
Recreational fishing	33.0	40.0	515,376

Value added taken from SwAM (2017), except for recreational fisheries that is based on unpublished SwAM data. Impacts and dependency on ecosystem services were assessed in the study

^a^Recreational fishing excluded

## Footnotes

1 Including all types of economic activities (e.g., restaurants, accommodation, tourist agencies) classified as tourism, within 1 km from the coast. This is how all European Union countries calculate the value of marine tourism, and that is the most precise method currently available.
