# Supplementary material for: A Model for Disentangling Dependencies and Impacts among Human Activities and Marine Ecosystem Services
Source: Environ Manage. 2020 Feb 27;65(5):575–86. doi: 10.1007/s00267-020-01260-1 (PMC7145787; doi:10.1007/s00267-020-01260-1)
Supplement: Supplementary file 1 — Supplement 1 [file 267_2020_1260_MOESM1_ESM.docx]

Supplement 1

*Table S1. The impact of activities on ecosystem services. Ecosystem services are indicated by their abbreviations provided in Table 2.*

| **Activity** | **RM1 BCC** | **RM2 PP** | **RM3 FWD** | **RM4 BDIV** | **RM5 HAB** | **RM6 RSIL** | **RM7 CA** | **RM8 SRET** | **RM9 EUT** | **RM10**  **BIOL** | **RM11**  **TOX** | **P1 FOOD** | **P2 RAW** | **P3 GEN** | **P4 CHE** | **P5 ORN** | **P6 ENRG** | **C1 RECR** | **C2 AEST** | **C3 SCIED** | **C4 CULH** | **C5 INSP** | **C6 NATH** | **Sum** |
| --- | --- | --- | --- | --- | --- | --- | --- | --- | --- | --- | --- | --- | --- | --- | --- | --- | --- | --- | --- | --- | --- | --- | --- | --- |
| **Land claim** | 1 | 1 | 1 | 1 | 2 | 1 | 0 | 1 | 1 | 0 | 1 | 1 | 0 | 0 | 0 | 0 | 0 | 1 | 1 | 0 | 1 | 0 | 1 | 15 |
| **Restructuring of seabed morphology** | 2 | 1 | 1 | 2 | 2 | 2 | 0 | 1 | 1 | 1 | 2 | 2 | 1 | 0 | 0 | 0 | 0 | 1 | 2 | 0 | 1 | 1 | 2 | 25 |
| **Extraction of minerals** | 1 | 1 | 1 | 1 | 1 | 1 | 0 | 1 | 1 | 1 | 1 | 1 | 2 | 0 | 0 | 0 | 0 | 1 | 2 | 0 | 1 | 1 | 2 | 20 |
| **Renewable energy generation** | 0 | 1 | 1 | 1 | 2 | 1 | 0 | 1 | 0 | 0 | 0 | 0 | 0 | 0 | 0 | 0 | 0 | 1 | 2 | 0 | 2 | 1 | 2 | 15 |
| **Nuclear power** | 1 | 1 | 1 | 1 | 1 | 1 | 0 | 1 | 0 | 0 | 1 | 1 | 1 | 1 | 0 | 0 | 0 | 1 | 1 | 0 | 1 | 1 | 2 | 17 |
| **Transmission (cables)** | 1 | 0 | 1 | 1 | 1 | 1 | 0 | 1 | 0 | 0 | 0 | 0 | 0 | 0 | 0 | 0 | 0 | 1 | 1 | 0 | 0 | 0 | 1 | 9 |
| **Fish and shellfish harvesting (prof.)** | 2 | 2 | 4 | 4 | 4 | 3 | 0 | 4 | 2 | 3 | 2 | 4 | 2 | 3 | 1 | 1 | 1 | 2 | 1 | 1 | 2 | 1 | 4 | 53 |
| **Hunting and collecting** | 0 | 0 | 1 | 2 | 1 | 1 | 0 | 0 | 0 | 0 | 0 | 1 | 0 | 0 | 0 | 0 | 0 | 2 | 0 | 0 | 0 | 1 | 1 | 10 |
| **Aquaculture** | 2 | 1 | 1 | 1 | 2 | 1 | 0 | 0 | 2 | 1 | 1 | 0 | 0 | 2 | 0 | 0 | 0 | 1 | 1 | 0 | 1 | 0 | 1 | 18 |
| **Agriculture** | 3 | 3 | 2 | 2 | 2 | 2 | 2 | 1 | 3 | 2 | 1 | 2 | 1 | 1 | 0 | 0 | 0 | 2 | 2 | 1 | 1 | 1 | 1 | 35 |
| **Forestry** | 3 | 3 | 2 | 2 | 2 | 2 | 2 | 1 | 2 | 2 | 1 | 2 | 1 | 0 | 0 | 0 | 0 | 2 | 2 | 1 | 0 | 1 | 2 | 33 |
| **Transport — infrastructure** | 1 | 1 | 1 | 2 | 2 | 1 | 1 | 1 | 2 | 2 | 1 | 2 | 1 | 0 | 0 | 0 | 0 | 2 | 2 | 0 | 1 | 1 | 2 | 26 |
| **Transport — shipping** | 1 | 1 | 1 | 2 | 2 | 2 | 1 | 2 | 2 | 2 | 2 | 1 | 1 | 1 | 0 | 0 | 0 | 2 | 2 | 0 | 2 | 1 | 2 | 30 |
| **Urban uses** | 1 | 1 | 1 | 1 | 2 | 1 | 1 | 1 | 2 | 1 | 1 | 2 | 1 | 0 | 0 | 0 | 0 | 1 | 2 | 0 | 2 | 1 | 2 | 24 |
| **Industrial uses** | 2 | 2 | 2 | 2 | 2 | 2 | 1 | 1 | 1 | 1 | 2 | 2 | 1 | 1 | 0 | 0 | 0 | 2 | 2 | 0 | 2 | 1 | 2 | 31 |
| **Waste treatment and disposal** | 2 | 3 | 2 | 2 | 2 | 2 | 0 | 1 | 2 | 2 | 3 | 3 | 2 | 1 | 0 | 0 | 0 | 2 | 2 | 0 | 0 | 1 | 2 | 34 |
| **Tourism and leisure infrastructure** | 1 | 1 | 1 | 1 | 2 | 1 | 0 | 2 | 1 | 1 | 1 | 2 | 1 | 0 | 0 | 0 | 0 | 1 | 2 | 0 | 1 | 1 | 2 | 22 |
| **Tourism and leisure activities** | 1 | 2 | 2 | 2 | 2 | 2 | 1 | 2 | 2 | 2 | 2 | 2 | 1 | 0 | 0 | 0 | 0 | 2 | 1 | 0 | 1 | 1 | 1 | 29 |
| **Fish and shellfish harvesting (recr.)** | 1 | 2 | 2 | 2 | 1 | 2 | 0 | 1 | 2 | 2 | 1 | 2 | 2 | 2 | 1 | 1 | 1 | 2 | 1 | 0 | 1 | 1 | 3 | 33 |
| **Security/defence, Military operations** | 0 | 0 | 0 | 0 | 1 | 0 | 0 | 1 | 0 | 0 | 1 | 1 | 0 | 0 | 0 | 0 | 0 | 1 | 1 | 1 | 1 | 2 | 1 | 11 |
| **Scientific and educational activities** | 0 | 0 | 0 | 1 | 0 | 0 | 0 | 0 | 0 | 0 | 0 | 0 | 0 | 0 | 0 | 0 | 0 | 0 | 0 | 0 | 0 | 0 | 0 | 1 |
| **Eutrophication** | 4 | 3 | 3 | 3 | 4 | 4 | 2 | 2 | 4 | 3 | 1 | 2 | 2 | 1 | 0 | 0 | 0 | 4 | 2 | 1 | 1 | 2 | 3 | 51 |
| **Toxic pollution** | 1 | 0 | 2 | 2 | 1 | 2 | 0 | 0 | 0 | 2 | 3 | 4 | 2 | 1 | 0 | 0 | 0 | 2 | 1 | 0 | 1 | 2 | 2 | 28 |
| **Climate change CO2** | 3 | 2 | 2 | 2 | 2 | 2 | 2 | 0 | 1 | 2 | 1 | 1 | 1 | 1 | 0 | 0 | 0 | 1 | 1 | 0 | 0 | 1 | 1 | 26 |
| **Climate change Temperature** | 2 | 3 | 2 | 2 | 3 | 2 | 4 | 2 | 1 | 2 | 1 | 2 | 2 | 1 | 0 | 0 | 0 | 1 | 1 | 0 | 1 | 1 | 1 | 34 |
| **Climate change Salinity** | 2 | 2 | 2 | 4 | 4 | 2 | 1 | 2 | 1 | 2 | 1 | 2 | 1 | 2 | 1 | 1 | 0 | 1 | 1 | 0 | 2 | 1 | 2 | 37 |
| **Sum** | 38 | 37 | 39 | 46 | 50 | 41 | 18 | 30 | 33 | 34 | 31 | 42 | 26 | 18 | 3 | 3 | 2 | 39 | 36 | 5 | 26 | 25 | 45 |  |
