# Supplementary material for: A Model for Disentangling Dependencies and Impacts among Human Activities and Marine Ecosystem Services
Source: Environ Manage. 2020 Feb 27;65(5):575–86. doi: 10.1007/s00267-020-01260-1 (PMC7145787; doi:10.1007/s00267-020-01260-1)
Supplement: Supplementary file 2 — Supplement 2 [file 267_2020_1260_MOESM2_ESM.docx]

Supplement 2

*Table S2. Activities’ dependency of ecosystem services. Ecosystem services are indicated by their abbreviations provided in Table 2.*

| **Activity** | **RM1 BCC** | **RM2 PP** | **RM3 FWD** | **RM4 BDIV** | **RM5 HAB** | **RM6 RSIL** | **RM7 CA** | **RM8 SRET** | **RM9 EUT** | **RM10**  **BIOL** | **RM11**  **TOX** | **P1 FOOD** | **P2 RAW** | **P3 GEN** | **P4 CHE** | **P5 ORN** | **P6 ENRG** | **C1 RECR** | **C2 AEST** | **C3 SCIED** | **C4 CULH** | **C5 INSP** | **C6 NATH** | **Sum** |
| --- | --- | --- | --- | --- | --- | --- | --- | --- | --- | --- | --- | --- | --- | --- | --- | --- | --- | --- | --- | --- | --- | --- | --- | --- |
| **Land claim** | 1 | 1 | 0 | 0 | 0 | 0 | 1 | 4 | 0 | 0 | 0 | 0 | 1 | 0 | 1 | 0 | 0 | 1 | 0 | 1 | 0 | 0 | 0 | 11 |
| **Restructuring of seabed morphology** | 1 | 1 | 0 | 0 | 0 | 0 | 0 | 4 | 1 | 0 | 2 | 0 | 0 | 0 | 0 | 0 | 0 | 1 | 0 | 1 | 0 | 0 | 0 | 11 |
| **Extraction of minerals** | 1 | 0 | 0 | 0 | 0 | 0 | 0 | 1 | 0 | 0 | 0 | 0 | 1 | 0 | 0 | 0 | 0 | 0 | 0 | 1 | 0 | 0 | 0 | 4 |
| **Renewable energy generation** | 0 | 0 | 0 | 0 | 0 | 0 | 1 | 0 | 0 | 0 | 0 | 0 | 0 | 0 | 0 | 0 | 1 | 0 | 0 | 1 | 0 | 0 | 0 | 3 |
| **Nuclear power** | 0 | 0 | 0 | 0 | 0 | 0 | 1 | 0 | 0 | 0 | 0 | 0 | 1 | 0 | 0 | 0 | 0 | 0 | 0 | 1 | 0 | 0 | 0 | 3 |
| **Transmission (cables)** | 0 | 0 | 0 | 0 | 0 | 0 | 0 | 0 | 0 | 0 | 0 | 0 | 0 | 0 | 0 | 0 | 1 | 0 | 0 | 1 | 0 | 0 | 0 | 2 |
| **Fish and shellfish harvesting (prof.)** | 1 | 3 | 4 | 4 | 4 | 2 | 1 | 1 | 4 | 4 | 2 | 4 | 2 | 1 | 1 | 0 | 1 | 0 | 0 | 2 | 1 | 1 | 1 | 44 |
| **Hunting and collecting** | 1 | 2 | 3 | 2 | 3 | 2 | 1 | 0 | 2 | 2 | 1 | 4 | 1 | 0 | 0 | 1 | 0 | 2 | 0 | 1 | 1 | 1 | 1 | 31 |
| **Aquaculture** | 1 | 0 | 1 | 1 | 1 | 1 | 0 | 0 | 1 | 1 | 2 | 2 | 1 | 1 | 0 | 0 | 0 | 0 | 0 | 2 | 1 | 0 | 0 | 16 |
| **Agriculture** | 0 | 0 | 0 | 0 | 0 | 0 | 2 | 0 | 0 | 0 | 0 | 0 | 0 | 0 | 0 | 0 | 0 | 0 | 0 | 0 | 1 | 0 | 0 | 3 |
| **Forestry** | 0 | 0 | 0 | 0 | 0 | 0 | 2 | 0 | 0 | 0 | 0 | 0 | 0 | 0 | 0 | 0 | 0 | 0 | 0 | 0 | 1 | 0 | 0 | 3 |
| **Transport — infrastructure** | 1 | 0 | 0 | 0 | 0 | 0 | 1 | 1 | 0 | 0 | 0 | 1 | 0 | 0 | 0 | 0 | 0 | 1 | 0 | 0 | 1 | 0 | 0 | 6 |
| **Transport — shipping** | 1 | 0 | 0 | 0 | 0 | 0 | 1 | 1 | 0 | 0 | 0 | 1 | 0 | 0 | 0 | 0 | 0 | 1 | 1 | 0 | 1 | 0 | 0 | 7 |
| **Urban uses** | 1 | 0 | 0 | 0 | 0 | 0 | 2 | 0 | 1 | 1 | 1 | 1 | 0 | 0 | 0 | 0 | 1 | 1 | 1 | 1 | 2 | 0 | 1 | 14 |
| **Industrial uses** | 1 | 0 | 0 | 0 | 0 | 0 | 1 | 0 | 0 | 0 | 0 | 0 | 1 | 0 | 1 | 0 | 1 | 0 | 0 | 1 | 0 | 0 | 0 | 6 |
| **Waste treatment and disposal** | 1 | 0 | 0 | 0 | 0 | 0 | 0 | 0 | 1 | 0 | 1 | 1 | 1 | 0 | 0 | 0 | 0 | 0 | 0 | 1 | 0 | 0 | 0 | 6 |
| **Tourism and leisure infrastructure** | 1 | 1 | 1 | 1 | 1 | 1 | 1 | 1 | 1 | 1 | 1 | 1 | 0 | 0 | 0 | 1 | 0 | 4 | 4 | 1 | 4 | 4 | 4 | 34 |
| **Tourism and leisure activities** | 1 | 2 | 1 | 2 | 2 | 1 | 1 | 0 | 2 | 0 | 1 | 1 | 0 | 0 | 0 | 1 | 0 | 4 | 4 | 0 | 4 | 4 | 4 | 35 |
| **Fish and shellfish harvesting (recr.)** | 1 | 3 | 3 | 4 | 3 | 2 | 1 | 0 | 1 | 1 | 1 | 2 | 0 | 0 | 0 | 1 | 0 | 4 | 2 | 1 | 2 | 4 | 4 | 40 |
| **Security/defence, Military operations** | 1 | 0 | 0 | 0 | 0 | 0 | 1 | 1 | 0 | 0 | 0 | 0 | 0 | 0 | 0 | 0 | 1 | 0 | 0 | 1 | 1 | 0 | 0 | 6 |
| **Scientific and educational activities** | 1 | 1 | 1 | 1 | 1 | 1 | 1 | 1 | 1 | 1 | 1 | 1 | 1 | 1 | 1 | 0 | 1 | 1 | 0 | 4 | 1 | 2 | 1 | 25 |
| **Sum** | 16 | 14 | 14 | 15 | 15 | 10 | 19 | 15 | 15 | 11 | 13 | 19 | 10 | 3 | 4 | 4 | 7 | 20 | 12 | 21 | 21 | 16 | 16 |  |
